# Grand Challenges in Allergen Immunotherapy

**DOI:** 10.3389/falgy.2021.710345

**Published:** 2021-08-05

**Authors:** Linda Cox

**Affiliations:** Retired, Casper, WY, United States

**Keywords:** allergen immunotherapy, allergic rhinitis, asthma, intralymphatic immunotherapy, epicutaneous immunotherapy, sublingual immunotherapy, subcutaneous immunotherapy

## Overview

Allergen immunotherapy (AIT) is a unique treatment for allergic disease in that it can provide both symptomatic relief and disease modification by modulating the allergen-induced immune response. A successful course of AIT can induce long-term clinical remission, prevent disease progression, and in some cases, it can be curative (e.g., Hymenoptera-induced anaphylaxis). It was first employed as a treatment for allergic rhinitis and asthma in the early 1900s, more than half of a century before Ishizaka identified immunoglobulin E as the reaginic antibody responsible for causing the allergic reaction (1). Jenner's successful work with the smallpox vaccine inspired, two English scientists, Noon and Freeman to apply the concept of injecting a causative substance to their “hayfever” patients (2, 3). In this case, the causative agent was not an infectious agent but rather a “pollen toxin” (3). Noon and Freeman theorized they could induce an immunity similar to the smallpox vaccine if they injected their patients with the “pollen toxin.” The specific causative allergen was confirmed by conjunctival provocation test with an extract prepared from the pollen, which was mixed in distilled water and then freeze-thawed and boiled. The CPT provided information on the degree of the patient's allergen hypersensitivity, which helped determine the AIT dosing regimen, particularly the starting dose. It was also used to monitor response to the patient's response to AIT. In their early clinical trials, Noon and Freeman observed that AIT's efficacy and safety was dose dependent. Noon summarized this observation in the following statements: “an overdose can induce a severe attack of hay fever lasting 24 hours” and the “sensibility of hay fever patients may be decreased by properly directed dosage” (2).

In retrospect, one could criticize the rationale of their theory as the smallpox vaccine was developed to prevent smallpox infection in individuals yet to be infected. Whereas, “hayfever” patients were already “infected” aka sensitized to the substance(s) they injected them with. In the ensuing 30 years after Noon and Freeman's initial publication on AIT use in allergic rhinitis, asthma, and food allergy, there was limited research, but continued use of AIT on both sides of the Atlantic. There were few advances in the understanding of how AIT works and what does it take to make it work (i.e., mechanisms and determinants of efficacy) until the latter part of the Twentieth century. Early studies suggested that AIT induced changes in the serum that could block the allergic reaction. In 1921, Fitzhugh and Lockey (4) demonstrated that anaphylaxis sensitivity could be passively transferred by the serum of the sensitive patient, a phenomenon that became known as the Fitzhugh and Lockey reaction) (5). In 1935, Fitzhugh and Lockey (4) demonstrated that patients treated with ragweed AIT could confer protection to unvaccinated ragweed allergic patients via serum transfer. This led to the theory that blocking antibodies were responsible for AIT's efficacy (5). It was not until Franklin and Lowell's research in the 1960s, that the dose-dependent efficacy observed by Noon and Freeman was confirmed (6). Their research also established that AIT efficacy was specific for the treated allergen (7). The mechanisms responsible for AIT's long-term efficacy continue to be elucidated and a better understanding of them is one of the “Grand Challenges” of AIT.

## Mechanisms

The immunological changes associated with effective AIT were not identified until the late-Twentieth century. Although earlier studies had found an association between AIT and decrease in allergen-specific IgE and rise in allergen-specific immunoglobulin G (IgG), the relationship with these allergen-specific immunoglobulin changes and AIT efficacy was not clear. In addition to changes in decreased allergen-specific IgE and increased allergen-specific IgG production, AIT has been associated with several other immunological events, which include: alterations in allergen-specific T- and B-cell cytokine responses, production of antibodies capable of blocking allergen presentation, thought to be of the IgG4 subset, reduction in tissue eosinophils and mast cells, and decreased basophil activation (8). Research has shown these immunological events take place at different time points in the AIT course. Mast cell and basophil desensitization are early events in AIT treatment. They are followed by the induction of IL-10 producing T and B regulatory cells that induce a B cell isotype switch that shifts the immunoglobulin production from IgE to IgG. There is also a shift in cytokine production which results in suppression of effector TH_1_ and TH_2_ cells (8). In a study that assessed grass-pollen allergic patients' skin test responses and cytokine production over a 1-year course of subcutaneous immunotherapy (SCIT), the increase in IL-10 production preceded the reduction in the late-phase skin response (9). This occurred early in the SCIT course (~2 weeks) and at a low dose of SCIT. Immunological events that occurred later in the AIT course (~6 weeks) include the changes in allergen-specific IgE and IgG discussed above. These events correlated with a reduction in the immediate skin test response. In another study examining the immunological changes associated with birch-pollen sublingual immunotherapy (SLIT), Bohle et al. found a similar rise in IL-10 production early in the course of treatment (4 weeks), which correlated with suppression of the peripheral blood mononuclear cell (PBMC) proliferation response to the treated allergen (Bet v 1), a related allergen (Mal d 1), and an unrelated antigen (tetanus toxoid) (10). Blockage with anti-IL-10 antibodies or removal of CD25(+) cells significantly increased the PBMC proliferation to all three antigens, i.e., the suppression induced by SLIT. At 52 weeks, the PBMC proliferation to the treated allergen, Bet v 1, remained suppressed, while the response to Mal d 1 and tetanus toxoid returned to pre-SLIT levels. Blockage with anti-IL-10 antibodies did not affect the suppressed Bet v 1-induced proliferation. At 52 weeks, a shift in mRNA cytokine expression suggested an immunological process other than IL-10. Compared with pre-SLIT values, there were significant reductions in 4 and IL-10 mRNA expression and a significant increase in IFN-gamma expression. Clonal deletion/anergy with an immune deviation of allergen-reactive T cells may be the mechanism responsible for AIT's sustained clinical efficacy and long-term immunological tolerance. The authors concluded that regulatory T-cells production of IL-10 production induces a non-specific antigen tolerance early in SLIT treatment, but this effect is not seen later in the treatment course. Immune deviation of allergen-specific T cells appears to be responsible for immunological tolerance seen later in the treatment course. In a double-blind, placebo-controlled study evaluating patients 1 year after discontinuation of a 3-year course of SCIT or placebo, SCIT-treated patients had significantly higher levels of IgE-blocking antibodies and allergen-specific IgG4 as compared with placebo (11). An earlier study that employed a rapid desensitization protocol demonstrated changes in allergen-specific IgG, which affected affinity for the allergen after 12 h of Hymenoptera immunotherapy (12).

These studies have provided some insights into the possible mechanisms responsible for AIT's efficacy, but they also make it apparent that there are several unanswered questions. Further research is needed to help us better understand “how AIT works,” so that it can direct research toward therapies that will make it “work better.” One of the primary aims of Frontier in Allergy's Allergen Immunotherapy section is to publish cutting-edge research to help us better understand AIT mechanisms.

## Allergy Diagnosis

Proper identification of the causative allergen(s) is essential to the development of effective AIT treatment. Incomplete identification and treatment of clinically relevant allergens have been identified as a reason for failure to improve with AIT (13). The allergy evaluation must include a thorough history and examination, which includes queries regarding the patient's exposures, triggers, and seasonal or other patterns. The clinical history should direct the allergy diagnostic work-up. The selection of allergens to be tested should be relevant for the patient's exposures. Allergy testing can be done *in vivo* (percutaneous and intradermal skin testing) or *in vitro* (serum allergen-specific IgE measurement via enzyme-linked immunoassays or other assays). There are circumstances in which the allergy skin and blood tests have their distinct advantages and limitations in the diagnostic process. A limitation of both methods is the testing reagents are generally crude allergen extracts, which are derived from natural sources (pollen, cat, dog, and Hymenoptera) or cultures (mold/fungi, cockroach, and house dust mite). The extracts contain complex heterogeneous mixtures of allergenic and non-allergenic proteins and macromolecules. The extract composition will vary with the extract processing method. In the United States, standardized grass-pollen and house dust mite SCIT extract potency is based on comparison with the Federal Drug Administration the Food and Drug Administration's (FDA's) Center for Biologics Evaluation and Research (CBER) reference control in an ELISA competitive assay, whereas standardized short ragweed and cat is based on measurement of the major allergen content. An allergen is defined as a “major allergen” if more than 50% of the allergic population produce specific IgE toward it. The term is a little misleading in that a “minor” allergens may be clinically significant for some individuals. Except for these four allergens and five Hymenoptera venoms, all other US SCIT extracts report content as either the weight of the source material extracted with a given volume of fluid (weight/volume) or protein nitrogen units (PNU) (14). Neither reporting method confers any direct or comparative information about the extract's composition or potency. Most European extract manufacturers express potency in the extract manufacturer proprietary units, which are generally based on titrated skin tests compared with an in-house reference control. Some manufacturers provide information about major allergen content, but few if any, provide a listing of the other identified allergens. None of the commercially available extracts provide a complete listing of the quantity of each of the allergenic components. Research suggests some extracts may be missing important allergens. For example, only one US dog extract contains significant amounts of the major dog allergen Can f 1 to achieve the projected therapeutic AIT dose (13). A study comparing five European dog extracts found considerable variability in the minor and major dog allergen contents (15). Most cat dander extracts do not contain significant amounts of albumin, to which 10% of the cat allergic population is sensitized to. There is evidence that Der p 23, a peritrophin-like protein, maybe a major HDM allergen (16, 17). Most commercial HDM extracts do not contain significant amounts of this protein (18). These are examples of how commercially available allergen extracts' limitations can impact the diagnosis and treatment of the allergic patient. To accurately diagnose and effectively treat allergic patients, the healthcare professional must know the composition of the allergen extracts used for testing and treatment. Unfortunately, this information is generally not easily available.

Another problem with allergy testing using currently available commercial allergen extracts is that they may identify IgE sensitization to panallergens, which may not clinically (e.g., Bet v 1 analogs, cross-reactive carbohydrate determinants). Molecular-based allergy diagnostics using biochip technology can measure sIgE antibodies against hundreds of allergenic molecules in a single assay (i.e., ISACC). This can provide a more precise map of a patient's allergen sensitization and better guide treatment (19).

Research to date has suggested molecular allergy diagnostic testing may be useful in determining what allergens should or should not be included in AIT treatment (20, 21). Little research has explored the impact of molecular-based allergy diagnostics on AIT outcomes, but there is some suggestion it can be useful in monitoring response to treatment (22).

Regardless of which allergy test method is used the results should always be interpreted in the context of the patient's clinical presentation, age, relevant allergen exposures, and the allergy tests' performance characteristics (e.g., sensitivity, specificity, reproducibility) (23). One of the greater challenges to transforming AIT from a ‘one extract fits all’ approach to one aligned with the principles of precision medicine is the availability of allergen extracts of known composition and potency for allergy diagnostic testing and AIT treatment. The Allergen Immunotherapy section of Frontiers in Allergy is committed to presenting research pertinent to advances in allergy diagnostic testing that result in more accurate and precise identification of causative allergen(s), an essential component of effective AIT.

## Biomarkers to Identify Ait Responders and Response to Treatment

Another significant AIT challenge is identifying biomarkers that can predict AIT treatment responders. Although many placebo-controlled trials have demonstrated significant clinical improvement during the first year of AIT, long-term efficacy appears to depend on a longer duration of treatment. The optimal duration of AIT is not known but it appears to be at least 3 years (24, 25). Practice guidelines suggest that AIT efficacy (or lack of) should not be accessed until at least a year after treatment commencement (13). Thus, AIT patients would have to complete at least 1 year of treatment to determine efficacy, and a multi-year course to appreciate the full and long-term benefits. This can be both time-consuming and costly for the patient. Research has yet to identify a marker that can invariably predict AIT responders (26). The pretreatment ratio of allergen-specific IgE to total IgE ratio (sIgE/tIgE) has been shown to correlate with clinical response to AIT in some studies (27, 28), but this was not confirmed in other studies (29–31). An European Academy of Allergy & Clinical Immunology's (EAACI) Task Force position paper concluded that there are no validated and generally accepted biomarkers that are predictive or indicative of the clinical response to AIT (32). The EAACI Task Force recommended exploration of allergen-specific sIgG4 as a biomarker for compliance. IgE-Facilitated Antigen Blocking (IgE-FAB) and sIgE/tIgE ratio assays were suggested as candidate biomarkers for clinical outcomes but the position paper stated: “more studies are needed to confirm and to interpret their association with the clinical response to immunotherapy and how they relate to persistence of clinical benefit after discontinuation of immunotherapy” (32).

## AIT Practical Considerations: Adherence, Costs, and Convenience

SCIT continues to be prescribed in a manner largely unchanged from Freeman's and Noon's protocol of administering unmodified extracts in increasing doses for weeks to months. The major disadvantage of SCIT is the relatively narrow margin between therapeutic efficacy and adverse side effects, Because of the risk of systemic allergic reactions that include rare life-threatening anaphylaxis, it is recommended that SCIT be administered in a medically supervised setting with an appropriate wait period (13). Efforts to develop safer and more effective AIT led to investigations with modified allergens and alternate delivery routes. Sublingual immunotherapy's (SLIT) safety and efficacy have been confirmed in multiple DBPC trials over the past 35 years. Its favorable safety profile allows for administration in a medically unsupervised administration, e.g., in the home setting. This adds a patient “convenience” factor to the advantage of ALIT over SCIT. SLIT dosing regimens are generally daily and the effective dose appears to be approximately 30 times the dose administered monthly with SCIT. Thus, the extract treatment costs of SLIT are greater than SCIT. Some studies suggest this is offset by SLIT's reduction in indirect costs, e.g., patient travel and/or lost work time to receive SCIT in a medical setting.

SLIT and SCIT appear to have comparable efficacy. However, there have been very few well-designed, controlled trials directly comparing their efficacy. Both routes require a commitment to several years of treatment and adherence to this is equally poor and similar to the poor compliance seen with long-term pharmacotherapy (33). Retrospective claims-based analysis studies, the rate of premature discontinuation of treatment was 45–93% of SLIT and 41–77% of SCIT patients (34). Cost and inconvenience were the most commonly cited reasons for discontinuation (34).

Two other alternative AIT routes that have demonstrated promising efficacy in the treatment of allergic disease are epicutaneous (EPIT) and intralymphatic immunotherapy (ILIT). EPIT involves the application of the allergen to the skin in the form of a patch. It has been studied in aeroallergen and food allergy. ILIT is a short-course treatment that involves three intratympanic allergen injections administered 1 month apart. Efficacy was demonstrated to be comparable to a 3-year course of SCIT in small open clinical trials (35). The short treatment course makes it one of the more attractive alternative AIT approaches, as this will likely address the poor adherence seen with SLIT and SCIT. However, large, randomized controlled trials are needed to establish its efficacy. ILIT and EPIT have not received regulatory authority approval in any country and are currently considered investigational.

In addition, to alternative routes, investigations aimed at developing better and more effective AIT have included adjuvants, peptides, recombinant allergen, and modified allergens. None to date have received regulatory authority approval.

## Conclusions

To align AIT with the principles of precision medicine, an approach that aims for specialized treatment regimens tailored to an individual's unique genetics, environment, and lifestyle, there are many unmet needs. These include access to comprehensive and accurate allergy diagnostic testing tools, a better understanding of the mechanisms, identification of biomarkers to predict and monitor response, and the development of safe, effective, affordable, and convenient treatments. The AIT section in Frontiers in Allergy will present research in these areas as well as on some practical applications such as cost-effectiveness, adherence, and other practical consideration in AIT treatment.

## Author Contributions

The author confirms being the sole contributor of this work and has approved it for publication.

## Conflict of Interest

The author declares that the research was conducted in the absence of any commercial or financial relationships that could be construed as a potential conflict of interest.

## Publisher's Note

All claims expressed in this article are solely those of the authors and do not necessarily represent those of their affiliated organizations, or those of the publisher, the editors and the reviewers. Any product that may be evaluated in this article, or claim that may be made by its manufacturer, is not guaranteed or endorsed by the publisher.

## Abbreviations

AIT: Allergen immunotherapy
CBER: Center for Biologics Evaluation and Research
CPT: Conjunctival provocation test
ELISA: enzyme-linked immunosorbent assay
FDA: Federal Drug Administration
IgE: Immunoglobulin E
IgG: Immunoglobulin G
SCIT: Subcutaneous immunotherapy
SLIT: Sublingual immunotherapy
US: United States.
